# Ligand-based discovery of coronavirus main protease inhibitors using MACAW molecular embeddings

**DOI:** 10.1080/14756366.2022.2132486

**Published:** 2022-10-28

**Authors:** Jie Dong, Mihayl Varbanov, Stéphanie Philippot, Fanny Vreken, Wen-bin Zeng, Vincent Blay

**Affiliations:** a Xiangya School of Pharmaceutical Sciences, Central South University, Changsha, P. R. China; b Université de Lorraine, CNRS, Nancy, France; c Laboratoire de Virologie, CHRU de Nancy Brabois, Vandoeuvre-lès-Nancy, France; d Department of Microbiology and Environmental Toxicology, University of California at Santa Cruz, Santa Cruz, CA, USA

**Keywords:** Coronavirus, ligand-based drug design, machine learning, cheminformatics, drug discovery

## Abstract

Ligand-based drug design methods are thought to require large experimental datasets to become useful for virtual screening. In this work, we propose a computational strategy to design novel inhibitors of coronavirus main protease, M^pro^. The pipeline integrates publicly available screening and binding affinity data in a two-stage machine-learning model using the recent MACAW embeddings. Once trained, the model can be deployed to rapidly screen large libraries of molecules *in silico*. Several hundred thousand compounds were virtually screened and 10 of them were selected for experimental testing. From these 10 compounds, 8 showed a clear inhibitory effect on recombinant M^pro^, with half-maximal inhibitory concentration values (IC_50_) in the range 0.18–18.82 μM. Cellular assays were also conducted to evaluate cytotoxic, haemolytic, and antiviral properties. A promising lead compound against coronavirus M^pro^ was identified with dose-dependent inhibition of virus infectivity and minimal toxicity on human MRC-5 cells.

## Introduction

With over 610 million reported human infections and 6.5 million deaths^1^, SARS-CoV-2, also denoted 2019-nCoV, is a positive-sense single-stranded RNA enveloped virus responsible for the ongoing COVID-19 pandemic. The virus mainly infects the respiratory system, where it can cause acute respiratory distress syndrome (ARDS) and fatal respiratory failure in some patients^2^, and it can also have long-lasting effects on other organs and systems, including long-term comorbidities (neurological disorders, memory loss, gastrointestinal distress, fatigue, insomnia, dyspnea) and post-acute sequelae of COVID-19 (PASC)^3–5^. Given the spread and severity of the disease, it is crucial to develop efficient treatments and rapidly available solutions that can supplement active immunisation efforts, which are still challenged by high viral transmissibility, re-infection, and immune escape variants^6^^,^^7^.

A variety of medicinal targets to fight infection by SARS-CoV-2 are being investigated^8^^,^^9^, and the main viral protease M^pro^ is particularly promising. After the virus infects and enters a human host cell, the two main ORF1a/b of its RNA genome first translate and express two polyprotein precursors (pp1a and pp1ab) with the help of the host cell machinery^10–14^. The polyprotein precursor undergoes intramolecular cleavage under the action of the main protease of SARS-CoV-2, M^pro^ (also known as 3C-like protease or 3CL^pro^), and the papain-like protease, PL^pro^, to produce multiple non-structural proteins (Nsps), Nsp1 to Nsp16. Some of the non-structural proteins produced participate in the production of viral subgenomic RNA encoding the four major structural proteins (Envelope/E protein, Membrane/M protein, Spike/S protein, and Nucleocapsid/N protein), which are needed to complete the reproduction and release of progeny viruses^10–14^.

At present, few antiviral drugs against SARS-CoV-2 are in or close to clinical use^15–17^. These include molnupiravir (Merck)^18^^,^^19^, Paxlovid (Pfizer)^20^, and PF-07304814 (Novartis)^21^. Paxlovid is a combination of the M^pro^ inhibitor nirmatrelvir (PF-07321332) and ritonavir (a CYP3A4 inhibitor that slows down clearance of nirmatrelvir)^20^. The potential of drug–drug interactions from ritonavir, however, may limit its use by many patients^22^. PF-07304814 is a prodrug under clinical trials that improves the pharmacokinetics of PF-00835231, another M^pro^ inhibitor^21^. Notwithstanding the advances, the possibility of resistance, the potential of combination therapies for treatment and prophylaxis, and the complexities of global logistics of the pandemic demand the development of additional antiviral therapies^23–25^. M^pro^ plays a vital role in mediating virus replication and transcription, and there is no homologous protein in humans. Besides, M^pro^ is highly conserved across different coronaviruses (alpha-, beta-, and gamma-coronaviruses)^26^^,^^27^ that might cause epidemics in the future. Thus, M^pro^ is an excellent target for the development of novel antiviral drugs against SARS-CoV-2, its variants, and other coronaviruses^16^^,^^28–31^.

Computational tools have played a prominent role in proposing potential M^pro^ inhibitors recently^32–40^. Most of these have leveraged structure-based drug design (SBDD) approaches, such as molecular docking, which rely on a structural model of M^pro^ to propose new ligands. However, few of these proposals have been experimentally validated. Besides, the protease is a complex target and computational and experimental efforts so far have faced low success rates^40–43^. On the other hand, the potential of ligand-based drug design (LBDD) has been little explored due to the scarcity of experimental data available early on during the pandemic. In the past months, some initiatives, such as NCATS’ COVID-19 OpenData Portal (https://opendata.ncats.nih.gov/covid19/) or PostEra’s Moonshot (https://covid.postera.ai/covid/activity_data), have been facilitating larger datasets that can enable this type of ligand-based approaches.

In this study, we explore a two-stage ligand-based drug design strategy for the discovery of novel M^pro^ inhibitors. We use MACAW embeddings, a recently proposed method to describe molecules computationally^44^, and build two predictive models on a curated compilation of experimental data. The models are applied in series to computationally assess hundreds of thousands of drug-like compounds. The most promising compounds are then sourced and evaluated experimentally for their ability to inhibit the viral protease and to arrest infection of human cells. The experimental results confirm the effectiveness of the virtual screening strategy, which can lead to rapid discovery of hit compounds with clinical potential against coronaviruses.

## Materials and methods

### Datasets

To model binding to SARS-CoV-2 M^pro^, we compiled two datasets from a variety of sources: a “regression dataset” and a “classification dataset”. For the regression dataset, we retrieved IC_50_ values from different studies^45–49^. IC_50_ values were converted to p*K*_i_ values using the Cheng-Prusoff equation, taking into account the substrate concentration and corresponding *K*_M_ values for each study (Equation (2)). The dataset was complemented with molecules from BindingDB (https://www.bindingdb.org/) with *K*_i_ values against M^pro^. As a result, we compiled 1716 molecules with their corresponding p*K*_i_ values (regression dataset).

(1) $${pK}_{i}=\;-\log_{10}(\frac{{IC}_{50}}{1+\frac{\left[S\right]}{K_{M}}})$$

To generate the classification dataset, we combined fluorescence primary HTS data from the Kuzikov and Zhu studies^46^^,^^47^. For the Zhu study, we considered as hits those molecules with p*K*_i_ >5.0 (labelled as 1). Molecules with lower pK_i_ values or with class 4 curves were considered non-binders (labelled as 0). For the Kuzikov study, we considered hits those molecules from the primary screening that were selected for subsequent hit confirmation, and non-hits otherwise. This corresponds roughly to the top 3% of the molecules tested. In addition, to complete the binary screening data, we looked at the pK_i_ data from the regression dataset compiled above (excluding the molecules from the Kuzikov and Zhu studies). We labelled the molecules as hits if they showed p*K*_i_ >5.0 and non-binders otherwise. Finally, we added molecules from BindingDB. Those molecules with p*K*_i_ >5.0 or pIC_50_ ≥5.0 were labelled as hits, whereas those with pK_i_ ≤5.0 or pIC_50_ <4.0 were labelled as non-binders. Note that, fromEquation (2), pIC_50_≤ p*K*_i_. This way, the classification dataset comprised 22 376 molecules labelled as hits (1) or non-binders (0).

Lastly, to discover new potential inhibitors of M^pro^, we compiled a virtual library composed of the Enamine Premium Collection (45 664 compounds), the Asinex Gold and Platinum Collections (261 120 compounds), and the DrugBank database of compounds (11 834 compounds)^50^. These are all lead- and drug-like molecules with favourable physicochemical properties (high Fsp3, low Log*P*, and MW, most of them satisfying Lipinski’s rule of 5).

### Hit classification

4261 molecules were downsampled from the screening dataset to attain a better balance between classes (1261 hits and 3000 non-hits). 20-D MACAW predictors were computed for each molecule. MACAW is a small-molecule embedding method that allows projecting molecules into a continuous, low-dimensional numerical space while extracting relevant molecular characteristics from the training dataset^44^. The distance between molecules in the embedding was defined by the combination of MACCS fingerprints and the Sokal similarity metric. 10% of the molecules were randomly held out as a test set. The MACAW predictors were then used to train a distance-weighted *k*-Nearest Neighbour classifier from scikit-learn 0.24.1^51^, with *k* = 10 and using a Euclidean distance metric (*p* = 2). Details are provided in the accompanying Jupyter Notebook 1.

### Affinity prediction

All molecules in the affinity dataset were projected into a 20-D embedding space using MACAW 1.0^44^. The distance between molecules in this case was specified by the combination of featurised Morgan fingerprints of radius 2 (featMorgan2) and the Tanimoto similarity metric. 10% of the molecules were randomly held out as the test set. The MACAW embeddings were used as inputs to a support vector regressor (SVR) from scikit-learn 0.24.1, with hyperparameters C = 3 and *ε* = 0.2. Details are provided in the accompanying Jupyter Notebook 1.

### ADMET property prediction

Poor pharmacokinetics is a key cause of drug candidate attrition. We used ADMETlab 2.0^52^ (https://admetmesh.scbdd.com) and SwissADME^53^ (http://www.swissadme.ch) to predict ADMET properties and help prioritise the compounds after the two-stage target binding predictions. We devised a custom scoring rule (ADMET_score) using KNIME (https://www.knime.com), which assigns specific weights to different ADMET properties based on the desirable range for each property. Higher ADMET_score values indicate a better predicted pharmacokinetic profile (see file SI01.xlsx).

### Molecular docking

Molecular docking was used to predict the binding mode of selected molecules against SARS-CoV-2 M^pro^ using the software MOE (Molecular Operating Environment) version 2019 (https://www.chemcomp.com). The crystal structure of SARS-CoV-2 M^pro^ in complex with N3 (PDB: 7BQY)^54^ was used as the template. The protein structure was first prepared by correcting amino acid residues, assigning ionisation states, and positioning hydrogens. Then the binding site of N3 was used as the pocket and each selected compound was docked against the pocket. Docking settings were chosen to reproduce the crystal pose of N3. In particular, the “Triangle Matcher” method with “London dG” score was used for placement, and the “Rigid Receptor” with “GBVI/WSA dG” score was used for replacement. 100 and 30 were set for the retained pose for the two stages. The best-scoring poses was considered the most likely binding mode. Further, to explore the potential of selected compounds binding to the II and III domains of the protein, we used the “site finder” to find a proper binding pocket there, and then the same pipeline was applied as above.

### Compound prioritisation

Compounds from the virtual screening libraries were prioritised for experimental testing based on their predicted classification probability of being a hit, their predicted binding affinity to M^pro^, and their predicted ADMET profile. In particular, compounds were considered of interest if they had a predicted hit probability ≥ 65% and a predicted p*K*_i_≥ 6.1. This led to 105 compounds (Supporting Information file SI01.xlsx). The compounds were then grouped in 15 clusters based on the pairwise similarity of their FragFp fingerprints using the software OSIRIS Datawarrior 5.2.1. At this stage, compounds were deemed most desirable if they exhibited predicted p*K*_i_≥ 6.5, hit probability ≥ 70%, ADMET_Score ≥25 (or coming from the DrugBank collection), and belonged to different clusters. The results were inspected, and 10 compounds were manually selected for experimental testing. The compounds were sourced from Topscience and Cayman and dissolved in DMSO as 10 mM stocks. All compounds were at least 90% as analysed by the supplier.

### Enzymatic assays

A FRET-based enzymatic cleavage test (Beyotime P0315M) was used to assess the inhibitory capacity of 10 compounds that had been prioritised computationally (Table 1). Each well of a 96-well fluorescence plate was dispensed with 92 μL of buffer, 1 μL M^pro^, and 5 μL of test compound in DMSO at a suitable concentration, such that the DMSO concentration was low and constant. Each test compound was tested at 10 different concentrations (0.01, 0.02, 0.05, 0.1, 0.5, 1, 5, 10, 15, and 20 μM final concentrations in the reaction mix). Lastly, 2 μL of the fluorogenic peptide substrate (MCA-AVLQSGFR-Lys(Dnp)-Lys-NH_2_) was introduced to each well to initiate the experiment. After adding the substrate as the final component, the reaction mix was incubated in the dark at 37 °C for 5 min. Fluorescence of each well was then measured at an excitation wavelength of 325 nm and an emission wavelength of 393 nm using a Thermo Varioskan LUX spectrophotometer. Ebselen was used as a positive control, which is known to bind covalently to the active site of M^pro^^55^. Cleavage of the fluorogenic substrate by M^pro^ leads to a separation of the FRET donor (Dnp) and acceptor (MCA) labels, leading to an increase in fluorescence from the acceptor label. Inhibitory compounds reduce the rate at which the fluorogenic substrate is cleaved and the fluorescence signal produced. The average fluorescence value of different wells can be recorded as RFU_blank_, RFU_100% enzyme_, RFU_positive_, and RFU_sample_, respectively, where RFU stands for Relative Fluorescence Units. The percentage of inhibition for each compound *i* was calculated as follows:

(2) $${\mathrm{Inhibition}}_{i}(\%)\;=\;\frac{{\mathrm{RFU}}_{100\%\mathrm{enzyme}}-{\mathrm{RFU}}_{i}}{{\mathrm{RFU}}_{100\%\mathrm{enzyme}}-{\mathrm{RFU}}_{\mathrm{blank}}}\times 100$$

**Table 1.** Information about the 10 compounds selected from the virtual screening of 408 935 lead-like molecules.

Id.	Structure	Catalog id. / CAS	Virtual screening	ADMET	
**1**	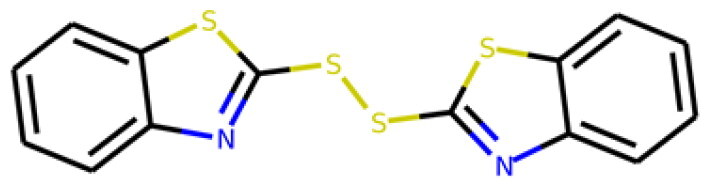	316789	p(hit) =1	MW = 331.96	HBD = 0
		120-78-5	p*K*i =6.453	log*P* = 5.298	HBA = 2
				TPSA = 25.78	QED = 0.449
**2**	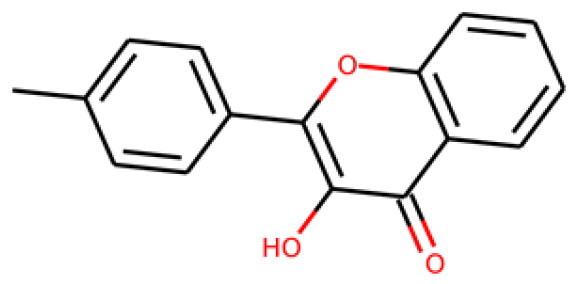	134210	p(hit) = 0.673	MW = 252.08	HBD = 1
		19275-68-4	p*K*i = 6.186	log*P* = 3.967	HBA = 3
				TPSA = 50.44	QED = 0.721
**3**	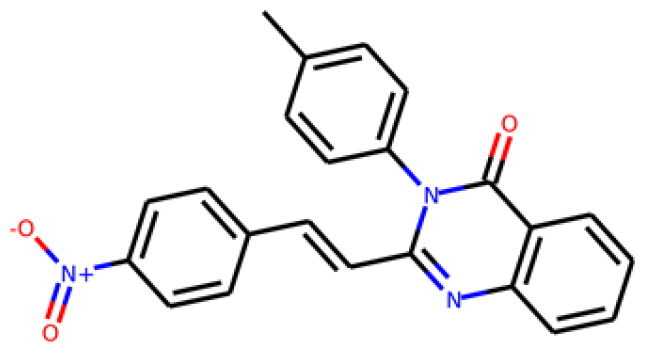	103542	p(hit) = 0.791	MW = 383.13	HBD = 0
		433262-70-5	p*K*i = 6.134	log*P* = 4.105	HBA = 6
				TPSA = 78.03	QED = 0.375
**4**	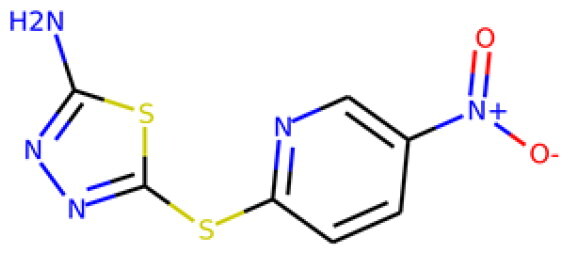	244527	p(hit) = 0.868	MW = 254.99	HBD = 2
		79134-17-1	p*K*i = 7.079	log*P* = 1.616	HBA = 7
				TPSA = 108.56	QED = 0.636
**5**	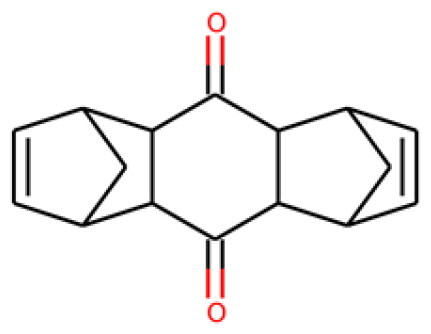	126615	p(hit) = 0.798	MW = 240.12	HBD = 2
		5439-22-5	p*K*i = 6.143	log*P* = 3.873	HBA = 2
				TPSA = 40.46	QED = 0.681
**6**	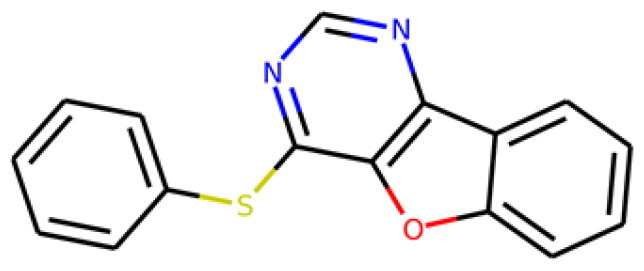	155928	p(hit) = 0.706	MW = 278.05	HBD = 0
		65023-97-4	p*K*i = 6.313	log*P* = 4.511	HBA = 3
				TPSA = 38.92	QED = 0.505
**7**	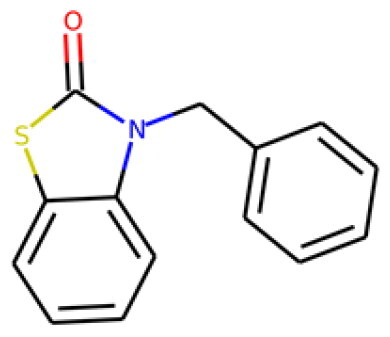	230318	p(hit) = 0.705	MW = 241.06	HBD = 0
		22291-74-3	p*K*i = 6.661	log*P* = 3.514	HBA = 2
				TPSA = 22	QED = 0.675
**8**	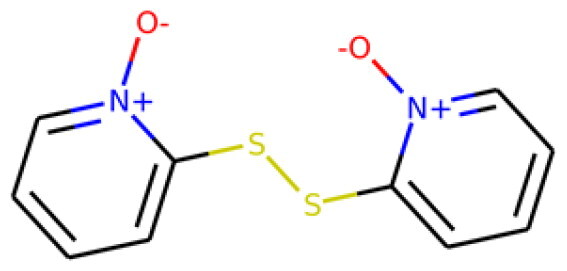	314478	p(hit) = 0.961	MW = 252	HBD = 0
		3696-28-4	p*K*i = 6.508	log*P* = 0.382	HBA = 4
				TPSA = 53.88	QED = 0.474
**9**	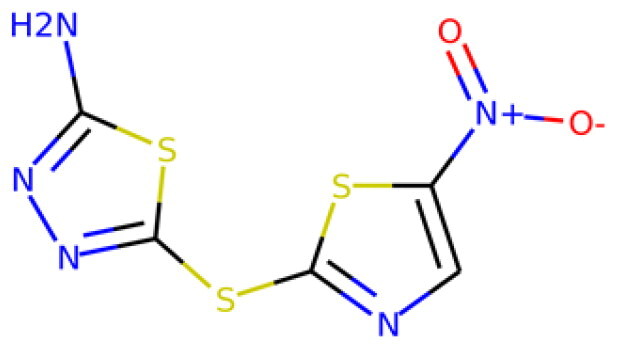	317502 40045-50-9	p(hit) = 1	MW = 260.94	HBD = 2
			p*K*i = 7.266	log*P* = 1.886	HBA = 7
				TPSA = 108.56	QED = 0.642
**10**	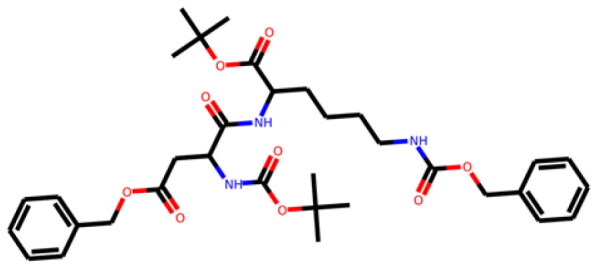	316703	p(hit) = 0.904	MW = 641.33	HBD = 3
		174630-04-7	p*K*i = 6.143	log*P* = 4.649	HBA = 12
				TPSA = 158.36	QED = 0.139

To exclude inhibitors possibly acting as aggregators, a detergent-based control was performed on selected compounds by adding 0.1% of freshly prepared Triton X-100 to the reaction mixture^54^. All experiments were performed in triplicate, and the experimental data was analysed using GraphPad Prism (https://www.graphpad.com).

### Cell model and culture

For virus propagation, MRC-5 pulmonary fibroblasts (ECACC, ref. 05090501) were grown in antibiotic-free Minimum Essential Media (MEM) with nonessential amino acids (Thermo Fisher Scientific), complemented with 10% foetal bovine serum (FBS, Eurobio), 2 mM glutamine (Sigma Aldrich), 1% nonessential amino acids (Gibco), and 1% sodium pyruvate (GE Healthcare). For the antiviral assays, the same medium was used, containing only 2% FBS.

### Virus

Human coronavirus hCoV-229 (PHE/NCPV 0310051v) was propagated and quantified in MRC-5 cells. Initial virus was titrated at 10^4^ IP/mL according to the Reed and Muench method^56^. All virus stocks were stored at −70 °C until used.

### Cytotoxicity

First, cellular toxicity was evaluated on MRC-5 in culture for 72 h in the presence of the selected compounds. Cells seeded the day before at 10 000 cells/well in a 96-well plate were treated with decreasing compound concentrations, from 100 to 3.12 μg/mL, in a culture medium containing only 2% FCS. Control wells of untreated or solvent-treated cells (DMSO), as well as blank cell-free wells, were added. After 72 h of incubation, cell viability was evaluated by the MTT test^57^.

### Cytopathogenic effect

The cells were seeded in 5 plates with 96 wells at 10 000 cells/well. After 24 h incubation, three dilutions of virus were carried out in cascade at 1/10, in culture medium (2% serum) with or without the test compound (at the highest concentration not inducing toxicity). The cell plates were emptied then the mats were treated with 100 μL of these dilutions (a column per condition, i.e. *n* = 8). An untreated control and a DMSO solvent control were included in each plate. The plates were incubated at 33 °C for 72 h. The cytopathic effect of the virus was observed under the microscope and the viral titres were determined according to the Reed and Muench method^56^. The plates were then stained with crystal violet and the cytopathic effect in each well was quantified thanks to the optical density at 540 nm. The percentage of cytopathic effect (% CPE) was calculated for treated and non-treated infected wells according to the formula:

(3) $${\mathrm{CPE}}_{i}(\%)\;=\;\frac{{\mathrm{OD}}_{\mathrm{sample},i}-\mathrm{mean}({\mathrm{OD}}_{\mathrm{blank}})}{\mathrm{mean}({\mathrm{OD}}_{\mathrm{control}})}\times 100$$

where the control was the non-treated and non-infected cells.

### Viral titres

The infectivity of HCoV 229E was determined by titration in triplicate on 96-well microtiter plates containing 100 μL of confluent MRC-5 cells. MRC-5 cells were added 100 µL from serial 10-fold dilutions of the virus from 10^1^ to 10^−8^ in MEM medium with 2% FCS. The infected cells were incubated at 37 °C in 5% CO_2_ for 72 h. The appearance of cytopathic effect (CPE) was recorded daily. The tissue culture infectious dose (50%) (TCID_50_), defined as the dilution of the virus required to infect 50% of the cell culture, was determined using the Reed and Muench method^56^ and expressed as TCID_50_/mL.

### Haemolysis

A haemolysis assay of the compounds was performed using human red blood cells (RBCs) to assess potential effects on the integrity of red blood cells. Selected compounds were diluted in PBS to relevant concentrations. RBCs were for 60 min at 37 °C. Two controls were used: a negative control containing RBCs and PBS, and a positive control in which RBCs were mixed with a product inducing haemolysis (Triton X-100 at 10%). After RBCs treatment, cells were centrifuged at 800 g for 5 min. The supernatant was recovered and measured with a spectrophotometer at 540 nm (Multiskan GO, Thermo Scientific, Saint Herblain, France).

## Results and discussion

### Virtual screens

MACAW is a recent computational tool that allows the featurisation of molecules for use in predictive machine-learning models, as well as the generation of molecules based on a predefined property specification^44^. In this work, we used the featurisation capabilities of MACAW. Low-dimensional MACAW embeddings can be directly used as inputs to machine-learning models, without the need of variable cleaning or feature selection steps that are often needed with conventional molecular descriptors.

**Figure 1.**
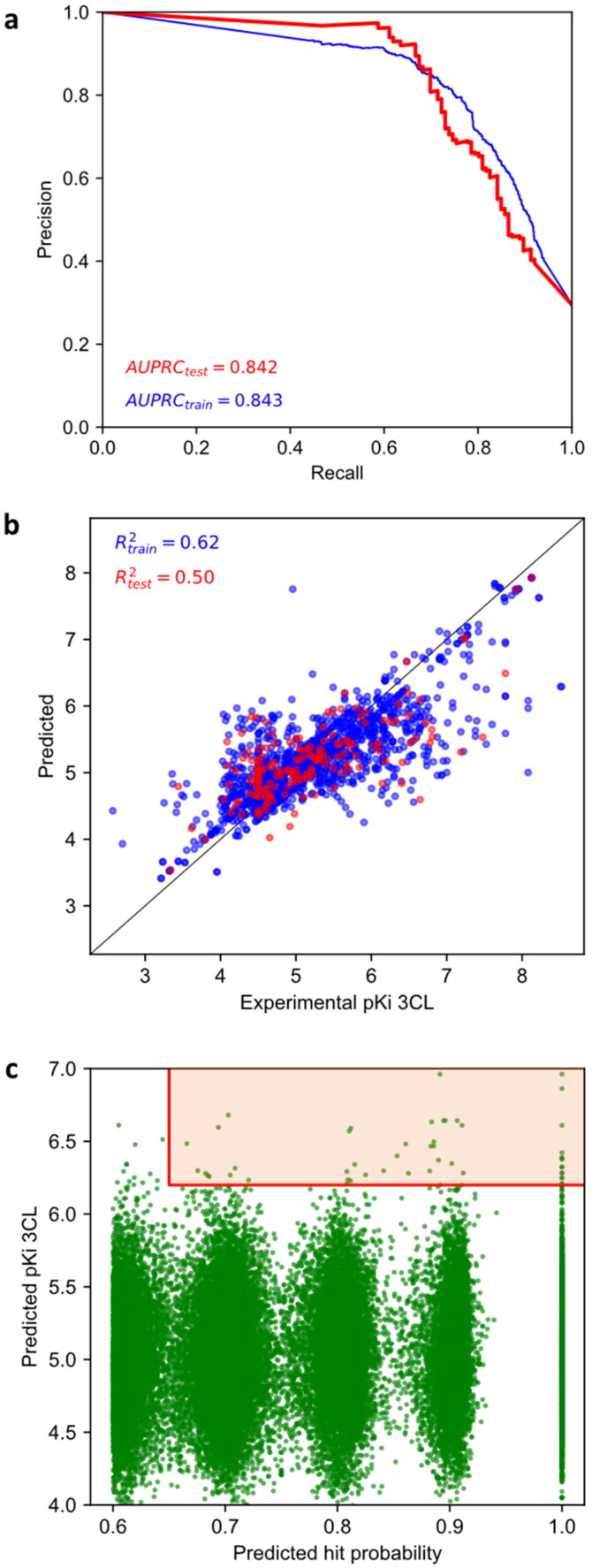
MACAW embeddings can help identify molecules able to bind to SARS-CoV-2 M^pro^. (a) Precision-recall curve of a kNN hit classifier trained on MACAW embeddings applied to a test set of molecules. (b) A SVR regressor was also trained to predict p*K*_i_ values for promising molecules. (c) We computationally screened a custom library of 408 935 lead-like molecules and prioritised 105 that both the classifier and the regressor considered promising (orange region). See Jupyter Notebook 1 for details.

A two-stage virtual screening of compounds was used. Firstly, a classifier trained on HTS data mainly rejected molecules unlikely to make promising hits. Given the large class imbalance in the classification dataset, we used a random downsampling strategy. As a classifier, we used a simple *k*-nearest neighbour classifier, which attained a notable AUPRC of 84% on the test set (Figure 1(a)). This case illustrates the usefulness of MACAW embeddings to rapidly deploy powerful classifiers.

Secondly, a regressor trained on affinity binding constants sought to prioritise potent molecules amongst those that may show some binding to the target. For this, a SVR model was trained on the respective MACAW embeddings for the regression dataset. In this case, the challenge is that the number of molecules for which quantitative affinity binding data is currently available is limited. Moreover, data is generally obtained in the form of IC_50_, which complicates comparison across different studies, as they often used different experimental conditions. The Cheng-Prusoff correction applied to the data can partially account for the difference (see “Methods” section), although it does not correct for other possible artefacts, like the potential dimerisation of M^pro^ *in vitro*^58^. Still, the regressor trained was able to achieve an *R*_train_ of almost 0.8 and an *R*_test_ above 0.7 (Figure 1(b)), which may contribute to increasing the enrichment provided by the virtual screening pipeline, which is the goal in this case.

With the two models (classifier and regressor) in place, we then interrogated a custom virtual library containing over 400 000 lead-like molecules. MACAW embeddings can be computed on a modest laptop in a few minutes and interesting molecules can be identified for further study (Figure 1(c)). The pipeline is elaborated in detail in the accompanying Jupyter Notebook 1.

From this library, 105 compounds were first extracted based on their predicted scores by the first and second models (see Supporting Information file SI01.xlsx). Some of the drugs highlighted in this list were identified as promising by early repurposing efforts. Notably, one of the highest ranked drugs in this list is ebselen, which has been reported as a potent inhibitor of M^pro^^55^. Another highly ranked compound is tideglusib, which has also been reported to inhibit the viral protease^59^. Thiram has also been shown to have antiviral activity against the virus, although possibly through another mechanism^60^. Taken together, these observations suggest that the two-stage screening pipeline may have potential in identifying novel small-molecule inhibitors of M^pro^.

Compounds were further shortlisted based on their predicted ADMET properties and chemical diversity. In particular, we kept compounds with pKi ≥6.5, hit probability ≥70%, and ADMET Score ≥25, which were further selected to increase diversity (see “Methods” section). The 10 compounds finally selected, along with some of their predicted properties, like the number of hydrogen bond donors (HBD) and acceptors (HBA) or the quantitative estimate of druglikeness (QED)^61^, are shown inTable 1.

### Enzymatic assays

**Figure 2.**
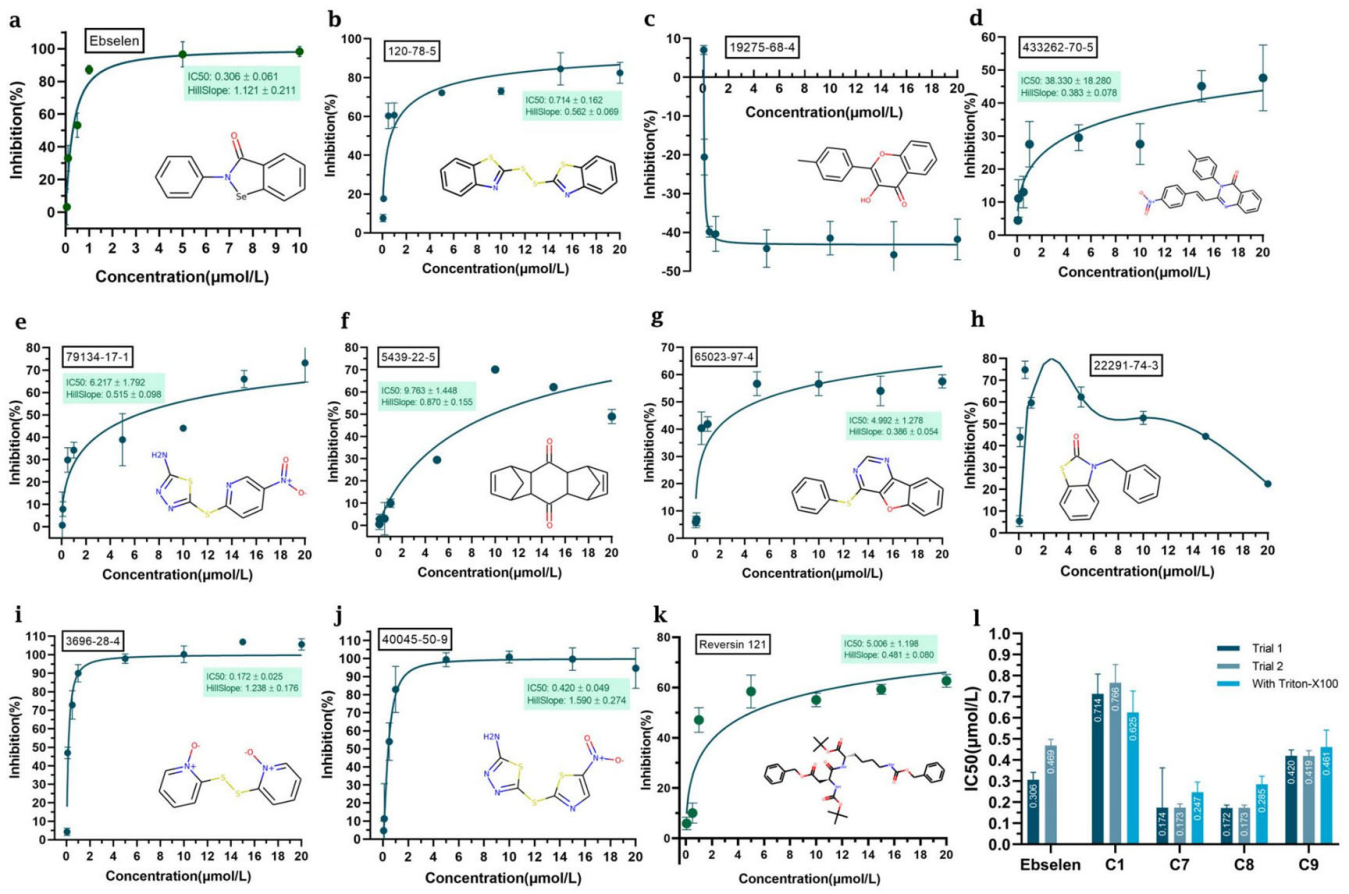
Selected compounds inhibit the activity of SARS-CoV-2 M^pro^. The hydrolytic activity of SARS-CoV-2 M^pro^ was measured in the presence of increasing concentrations of different test compounds. (**a**) Ebselen. (**b**) Compound **1**. (**c**) Compound **2**. (**d**) Compound **3**. (**e**) Compound **4**. (**f**) Compound **5**. (**g**) Compound **6**. (**h**) Compound **7**. (**i**) Compound **8**. (**j**) Compound **9**. (**k**) Compound **10**. (**l**) IC_50_ values for Ebselen, Compound **1**, Compound **7,** Compound **8**, and Compound **9**. The dose-response curves and IC_50_ values were determined by nonlinear regression. All data are shown as mean ± SEM, *n* = 3 biological replicates.

We evaluated the ability of the compounds shortlisted to inhibit the viral protease in an *in vitro* FRET-based assay using recombinant M^pro^. Active M^pro^ can cleave the labelled peptide substrate, leading to a fluorescent signal. If a compound that inhibits the proteolytic activity is present, the fluorescence signal will be diminished. Out of the 10 selected compounds, 8 have a clear inhibitory effect on M^pro^, with half-maximal inhibitory concentration values (IC_50_) in the range 0.18–18.82 μM (Figure 2). Among them, compounds 2 and 7 showed atypical inhibition results. Compound **8** (dipyrithione) displayed the strongest inhibitory effect in this assay, with an IC_50_ value of 0.18 μM. Notably, this value is even lower than that for the positive control with ebselen. Compounds **1** and **9** also displayed relatively low IC_50_ values of 0.71 and 0.42 μM, respectively. Interestingly, compound **7** seemed to be highly potent at low concentrations, although the fluorescent signal increased at high compound concentrations. Thus, we decided to not discard it and keep it in our pipeline instead.

**Figure 3.**
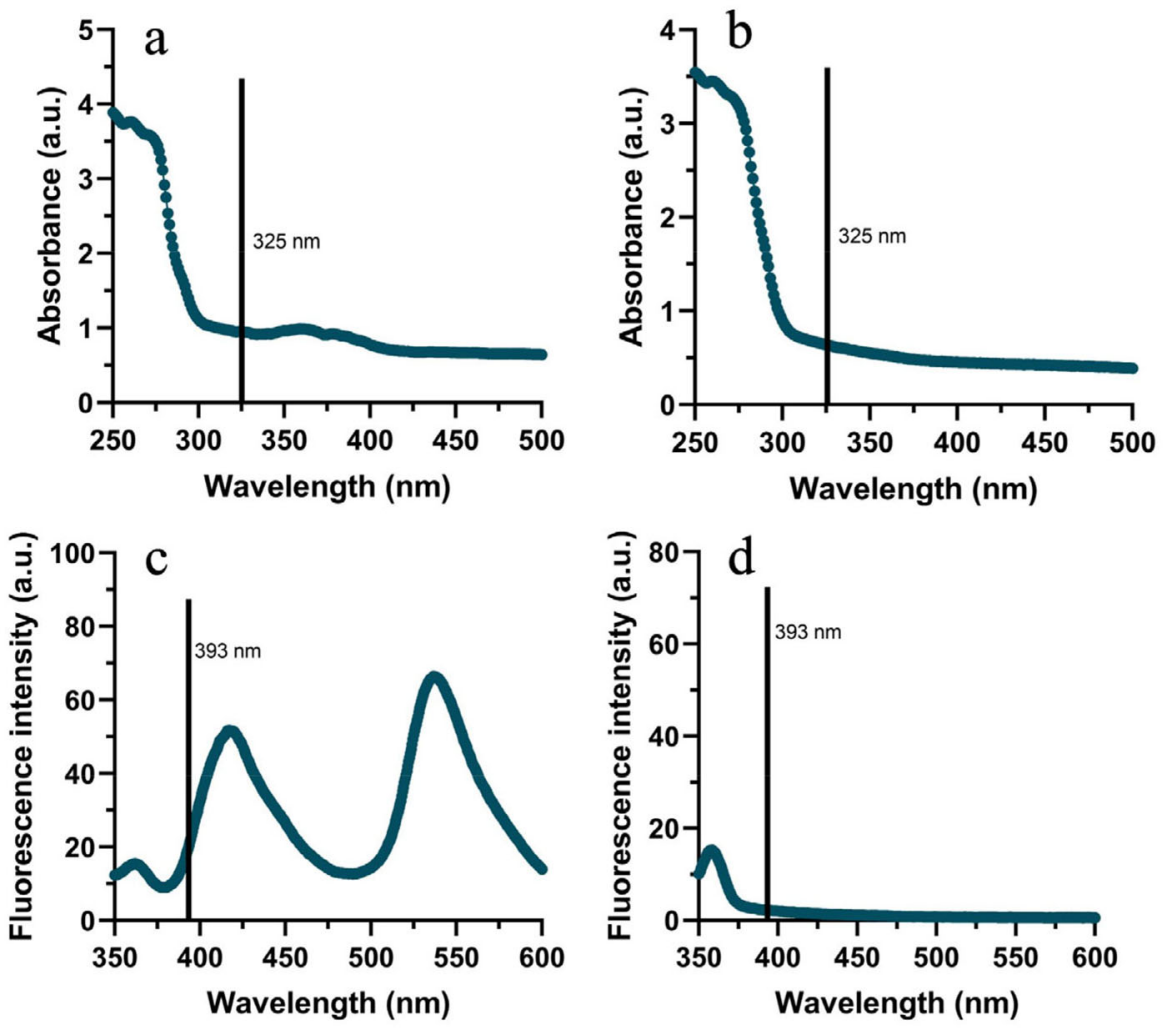
Spectra of compounds 2 and 7. UV-vis absorption spectra of compound **2** (a) and compound **7** (b). Fluorescence emission spectra of compound **2** (c) and compound **7** (d) at an excitation wavelength of 325 nm.

**Figure 4.**
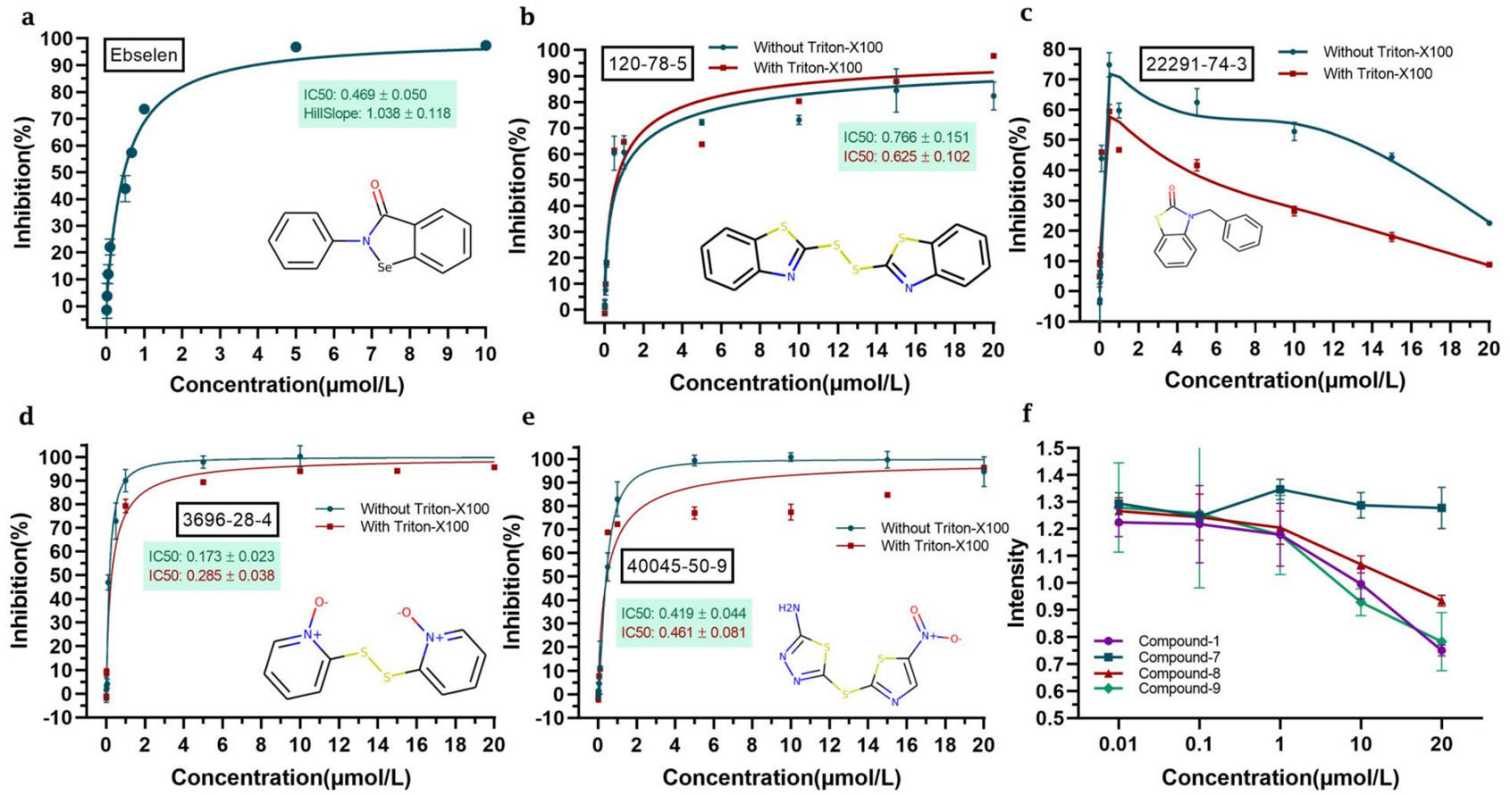
Addition of detergent does not affect the inhibition of SARS-CoV-2 M^pro^ by selected compounds. (**a**) The hydrolytic activity of SARS-CoV-2 M^pro^ was measured in the presence of increasing concentrations of ebselen. (**b–e**) IC_50_ values were determined in the presence and absence of 0.1% Triton X-100. (**b**) Compound **1**. (**c**) Compound **7**. (**d**) Compound **8**. (**e**) Compound **9**. (**f**) Autofluorescence of selected compounds at different concentrations. All data are shown as mean ± SEM, *n* = 3 biological replicates.

To analyse the potential effect of autofluorescence, UV-vis absorption spectra and fluorescent spectra of compounds **2** and **7** were measured (Figure 3). FromFigure 3(a,c), we can see that compound **2** had some absorption at 325 nm (the excitation wavelength used in the inhibition assay) and a significant fluorescence emission at 393 nm (the emission wavelength corresponding to the labelled substrate). Thus, autofluorescence may contribute to the atypical inhibition curve observed for compound **2** (Figure 2(c)). From Figure 4(b), we see that compound **7** has very weak absorption at 325 nm and it showed no fluorescence. This indicates that the inhibition curve for compound **7** is not affected by autofluorescence and other phenomena must explain its unusual trend.

On the other hand, upon addition of 0.1% Triton X-100 detergent, the results for compounds **1**, **7**, **8**, and **9** did not change significantly, confirming that they do not inhibit the viral protease by inducing aggregation (Figure 4). Compounds **1**, **7**, **8**, and **9** were thus selected for further study.

### Cellular assays

**Figure 5.**
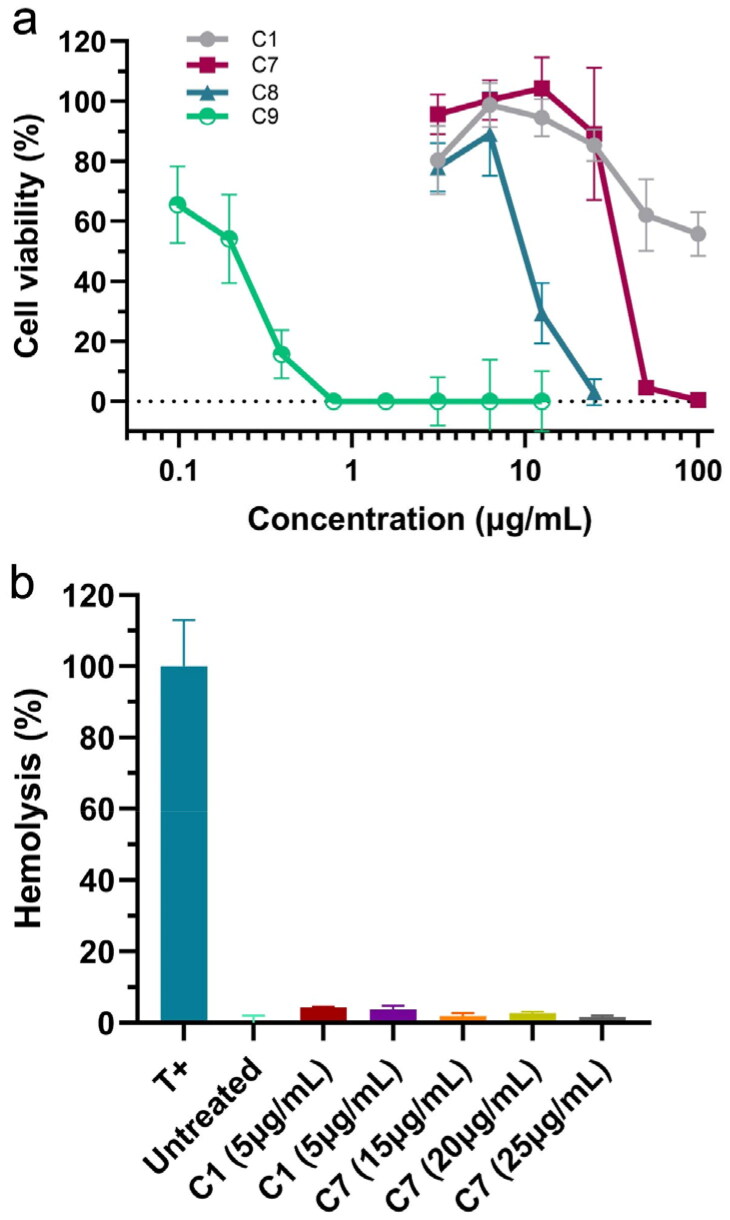
(a) Evaluation of cytotoxicity of compounds **1**, **7**, **8**, and **9** on MRC-5 cells at 72 h post-treatment by MTT test, *n* = 3. (b) Effects of compounds 1 and 7 on the haemolysis of red blood cells compared to a positive control (T+, 10% Triton X-100) and a negative control (Untreated), *n* = 3.

The cellular toxicity of compounds **1**, **7**, **8**, and **9** was evaluated on MRC-5 cells in culture for 72 h at varying concentrations, using cellular viability as a proxy (see “Methods” section). The results, shown in Figure 5, revealed that compound **9** is toxic. Compound **8** had limited toxicity, whereas compounds **1** and **7** showed minimal toxicity. The ADMET predictions in this work failed to flag compound **9** as toxic, indicating that it might be beneficial to conduct additional toxicity predictions *in silico* before prioritising compounds. In particular, nitroaromatic groups often display toxicity^62^. Interestingly, compound **9** was recently given the name “halicin” and found to have antibiotic properties through an unusual membrane associated-mechanism of action^63^. It is possible that this mechanism is toxic to some human cells as well. Our results thus clearly show that halicin can be highly toxic to human cells. Furthermore, pyrithione zinc complexes, related to compound **8**, have been very recently reported to be inhibitors of another viral protease, PL^pro^^64^.

**Figure 6.**
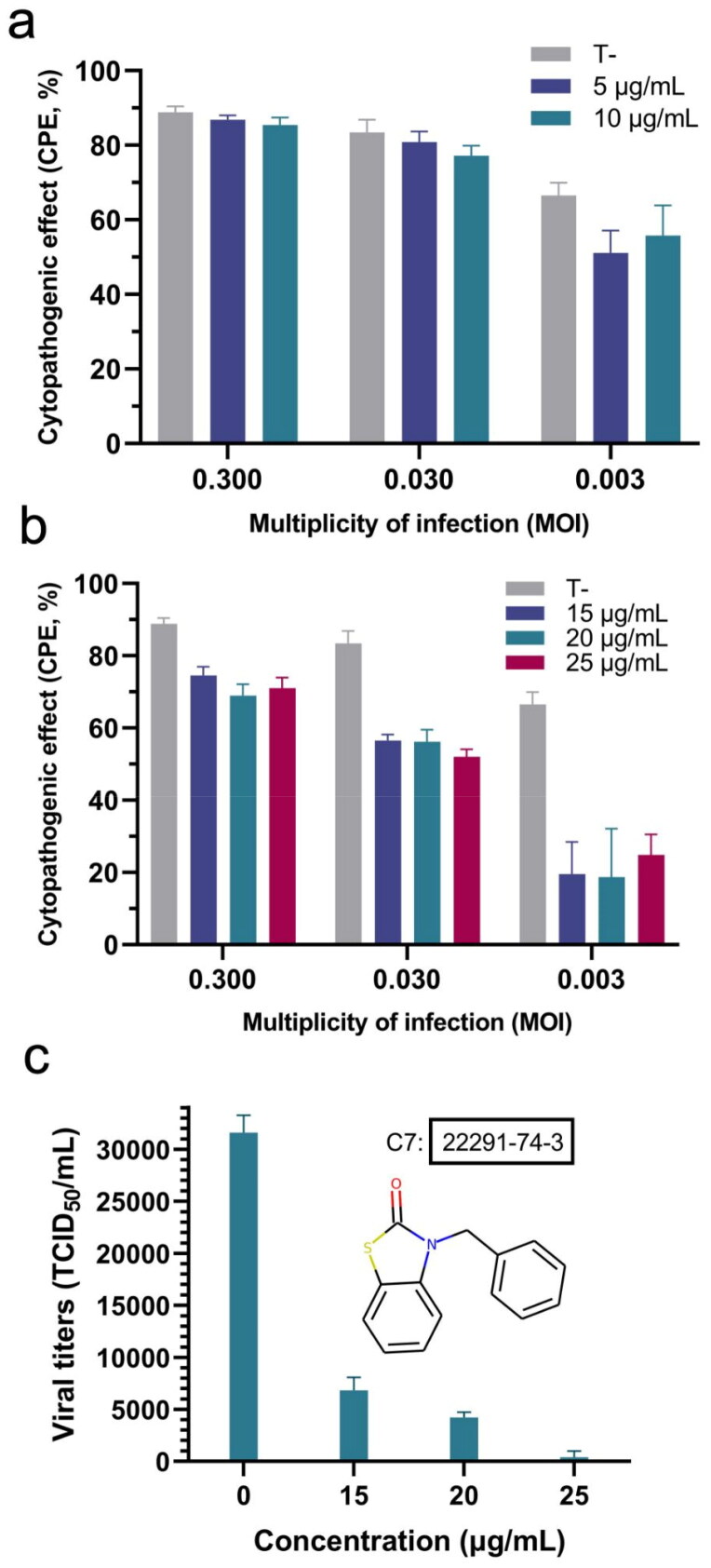
Evaluation of antiviral activity of compounds **1** and **7**. Effect of (a) compound **1** and (b) compound **7** on the cytopathogenic effect by coronavirus hCoV-229 at different viral loads, *n* = 3. (c) Tissue culture infectious dose (50%) (TCID_50_), defined as the dilution of the virus required to infect 50% of the cell culture, in the presence of different concentrations of compound **7**, *n* = 3.

Compounds **1** and **7** were also evaluated for their ability to inhibit the cytopathogenic effect of the virus (see Methods). The concentrations for this assay were selected based on the cytotoxicity results inFigure 5. The results for compound **1** (Figure 6(a)) show that it has no inhibitory effect on the cytopathogenic effect (CPE) of the virus at high multiplicity of infection (i.e. high viral load). Nonetheless, it may elicit some reduction in CPE at a low multiplicity of infection (MOI) of 0.003. Notably, compound **7** shows a very significant reduction in CPE across all the viral doses tested (Figure 6(b)). We evaluated the effect of compound **7** on viral titres (Figure 6(c)), confirming that the reduction in the cytopathic effect is associated with a substantial reduction in the viral load. This would be consistent with the main mechanism of action of compound **7** being the inhibition of M^pro^. Compound **7** thus appears as a promising antiviral hit compound against SARS-CoV-2, warranting further studies and optimisation efforts. Of note, 2(*3H*)-benzoxazolone and bioisosteres like 2(*3H*)-benzothiazolone in compound **7** are considered a “privileged scaffold”, which has been used in commercial drugs such as Benzolone, Paraflex, Vinizene, and Tiaramide^65^.

### Molecular docking results

**Figure 7.**
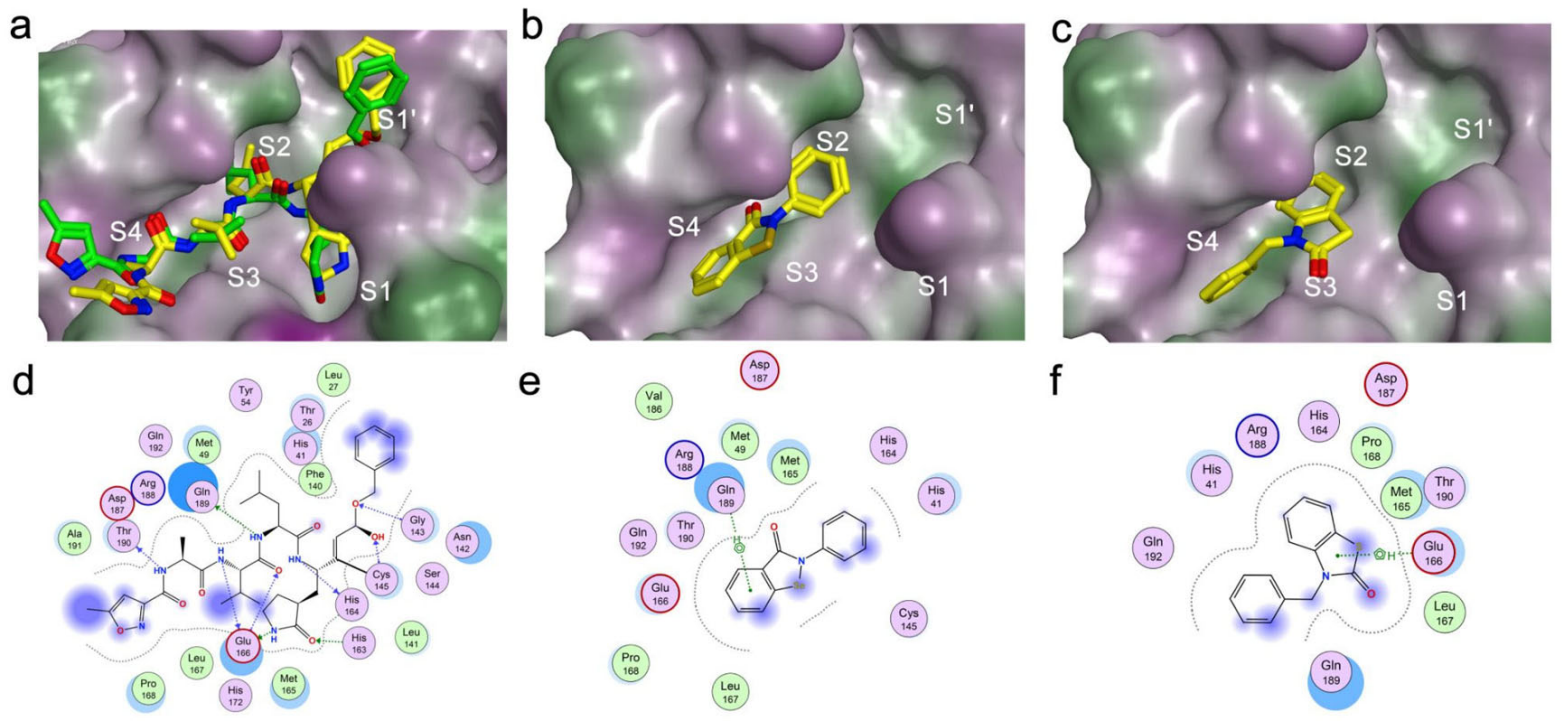
Docking results of N3, ebselen, and compound **7** against the catalytic site of M^pro^. (a) Highest scoring pose (yellow) and the crystallographic N3 pose (green). (b) Highest scoring pose of ebselen. (c) Highest scoring pose of compound **7**. (d–f) 2D interaction maps for the highest scoring poses of N3, ebselen and compound **7** with M^pro^, respectively.

We attempted to predict the potential binding pose of compound 7 on M^pro^ using molecular docking. Previous studies highlighted that the catalytic site of SARS-CoV-2 M^pro^ includes the regions S1′, S1, S2, S4, and the surface depression S3^54^^,^^66^, depicted in Figure 7. Since the complex structure of inhibitor N3 and M^pro^ has been resolved^54^, we first optimised the docking parameters to recapitulate the native pose of the inhibitor N3 (RMSD = 2.15 Å,Figure 7(a, d)). After optimising the docking parameters, we docked the positive control drug ebselen and compound **7**. The resulting docking poses and ligand interaction maps are shown inFigure 7(b–f).

From the docking results, we can see that ebselen and compound **7** may have similar binding poses. They both would occupy the S2 and S4 sites. Ebselen would form a pi–H interaction with residue Gln189, while compound **7** would form a pi–H interaction with Glu166. Actually, the second ranked pose of compound 7 also showed a pi–H interaction with Gln189, albeit with a poor docking score. This suggests that compound **7** may adopt a similar binding conformation to ebselen.

Given its small size, ebselen cannot form extensive interactions like N3, which occupies the entire S1 to S4 sites and obtains a high docking score, and yet ebselen still has high inhibitory activity. This is possible because ebselen can donate a selenium atom after a ring-opening reaction, forming a covalent bond and blocking the histidine-Cys catalytic dyad, thus acting as a covalent inhibitor. Although the docking of compound **7** suggests that it may have similar interactions as ebselen and its five-membered ring might also open, further studies are necessary to evaluate this possibility.

**Figure 8.**
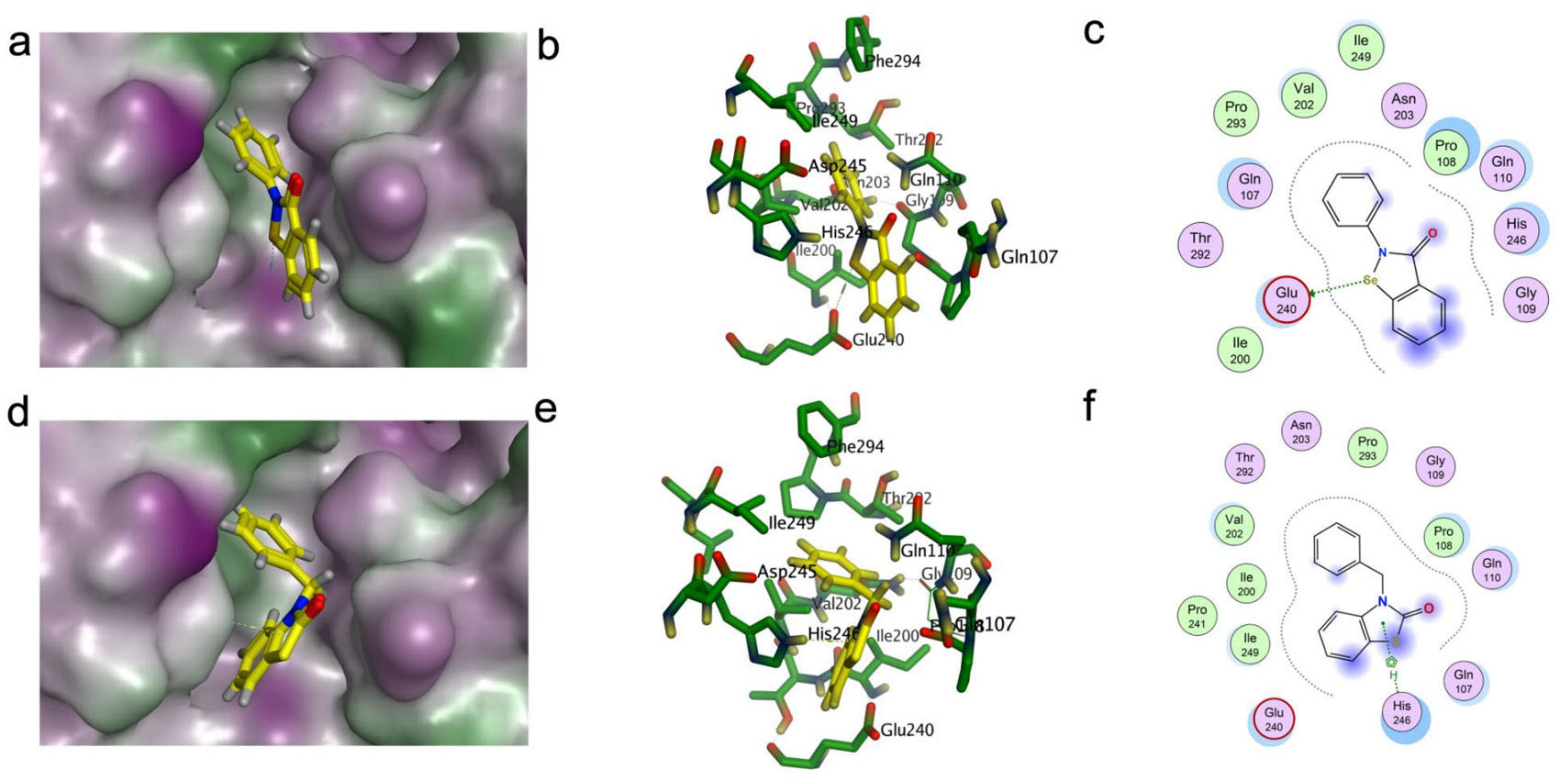
Docking results of ebselen and compound **7** in the region between domains II and III of M^pro^. The best pose of ebselen (a) and compound **7** (d) in that site. 3D visualisation of the key residues involved in the interaction between M^pro^ and ebselen (b) or compound **7** (e). 2D interaction maps for the best poses of ebselen (c) and compound 7(f) on M^pro^.

Recently, another study has shown that ebselen can bind not just to the catalytic site of SARS-CoV-2 M^pro^, but also to between the domains II and III of the protein, where it can act as an allosteric regulator^67^. In fact, allosteric inhibition of M^pro^ is increasingly recognised as a promising treatment modality^68^. This led us to reconsider the *in vitro* binding results above (Figure 2(h)). Compound **7** showed high inhibitory activity at low concentration, but its inhibition activity changed significantly at higher concentration. A possible explanation could be that compound **7** also acted as an allosteric modulator of the enzyme. Thus, we conducted a second docking study of ebselen and compound **7** against the site between domains II and III. The results are shown in Figure 8. Ebselen would form a hydrogen bond between its selenium atom and Glu240. It would also establish hydrophobic contacts with Gln107, Pro108, Gln110, Ile200, Val202, His246, Ile249, Thr292, and Phe294. These interactions are in good agreement with the findings of Menéndez et al.^67^. The molecular dynamics simulations by the researchers point to highly dynamic hydrogen bonds between ebselen and the side chains of Gln107, Gln110, and His246 side. FromFigure 8(b) we can see the oxygen atom approaching Gln107 and Gln110, likely forming hydrogen bonds under dynamic conditions. Compared to ebselen, compound **7** obtained a better docking score (0.4 kcal mol^−1^ lower) and a very similar interaction profile (Figure 8), supporting a possible effect in this allosteric site. Inspection of the electrostatic surface potential of the two pockets using the APBS formalism^69^ reveals some ressemblances between the sites (Supporting Information Figures S1 and S2), suggesting that both might participate in binding.

In summary, compound **7** showed a better binding potential than ebselen in both two sites of the viral protein given its docking scores and interaction profiles. Our results thus warrant further studies on the inhibitory mechanism of compound **7**.

## Conclusion

Viruses leverage biochemical machinery of host cells, which makes the design of viral inhibitors a challenging problem. Viral inhibitors would be greatly beneficial to complement vaccination approaches, particularly in the context of the current SARS-CoV-2 pandemic. The resolution of SARS-CoV-2 protein structures enabled the structure-based design of potential inhibitors of the main protease, M^pro^, as well as other viral targets. However, these approaches had limited success. The application of machine-learning ligand-based approaches is potentially more accurate, but it is thought to require large training datasets, which may not be available in a new epidemic. In this work, we show that a machine-learning ligand-based strategy to design small-molecule inhibitors of the coronavirus main protease can be useful even with limited data. We demonstrate a two-stage computational strategy based on the recent MACAW embeddings, which leverages both screening and affinity binding measurements publicly available to increase the probability of success. The pipeline can be used to make predictions for large libraries of compounds *in silico*. Out of 10 compounds selected with this pipeline, as many as 8 showed a clear inhibitory effect on M^pro^ when tested *in vitro*, with half-maximal inhibitory concentration values (IC_50_) in the range 0.18–18.82 μM. Additional assays were conducted to evaluate the cytotoxicity, haemolysis potential, and antiviral activity of selected compounds on MRC-5 cells. The results highlight the importance of considering ADMET properties early on in drug discovery. Most importantly, we identify compound **7**, 3-benzyl-1,3-benzothiazol-2-one, as a promising novel candidate against coronaviruses, demonstrating the utility of MACAW embeddings for molecular design.

## Supplementary Material

Supplemental Material
