# Supplementary material for: Ligand-based discovery of coronavirus main protease inhibitors using MACAW molecular embeddings
Source: J Enzyme Inhib Med Chem. 2022 Oct 28;38(1):24–35. doi: 10.1080/14756366.2022.2132486 (PMC9621234; doi:10.1080/14756366.2022.2132486)
Supplement: Supplemental Material [file IENZ_A_2132486_SM9162.pdf]

SUPPORTING INFORMATION

Ligand-based discovery of coronavirus main protease inhibitors using MACAW molecular embeddings

Jie Dong[a], Mihayl Varbanov[b,c], Stéphanie Philippot[b], Fanny Vreken[b], Wen-bin Zeng[a], Vincent Blay[d,]*

[a] Xiangya School of Pharmaceutical Sciences, Central South University, Changsha, 410013, P. R. China.

[b] Université de Lorraine, CNRS, L2CM, F-54000 Nancy, France.

[c] Laboratoire de Virologie, CHRU de Nancy Brabois, F-54500 Vandœuvre-lès-Nancy, France.

[d] Department of Microbiology and Environmental Toxicology, University of California at Santa Cruz, Santa Cruz, CA, 95064, USA. vroger@ucsc.edu

ORCID: 0000-0001-9602-2375 (V.B.), 0000-0002-3324-9000 (J.D.).

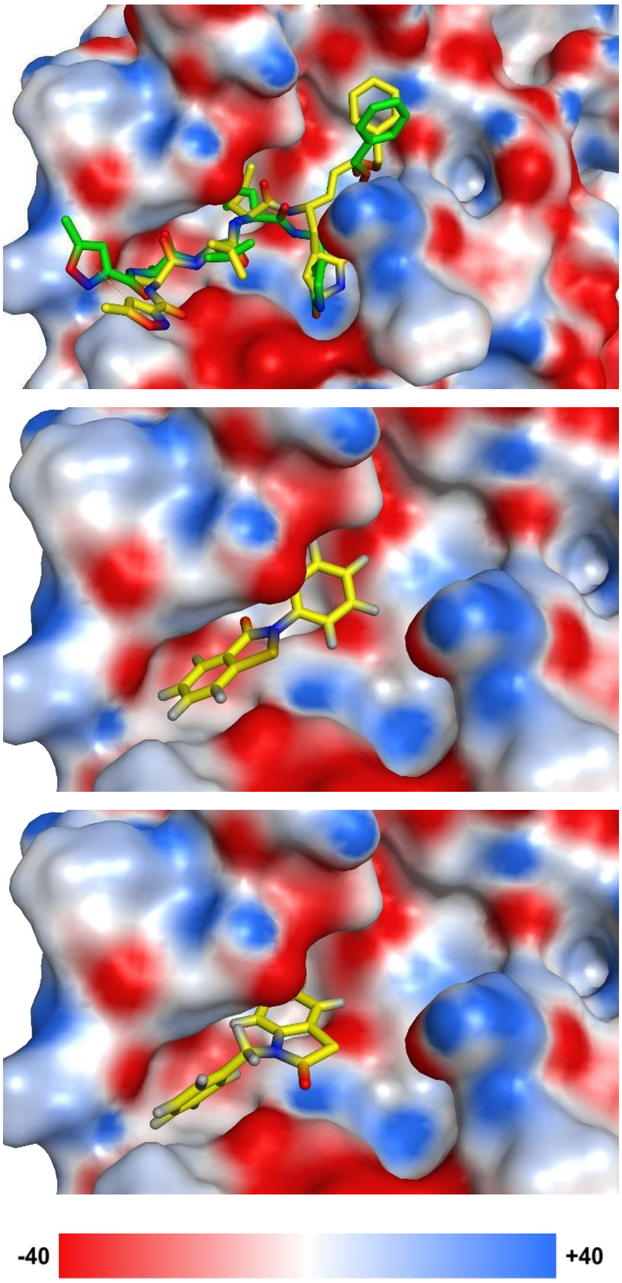

Figure S1. Docking results of N3 (top), ebselen (middle), and compound **7** (bottom) against the catalytic site of M^pro^. The protein is colored based on the electrostatic surface potential, computed using MOE 2019. The charge density is obtained by solving the Poisson-Boltzmann equation for the system. Significant patches are established by cutting the surface along iso-contour lines of absolute field value equal to 40 kcal mol^{-1} C^{-1}, keeping regions above. A default minimal patch area of 40 $Å^2$ filters out smaller, less relevant, patches. Potential values outside the range are clipped to the chosen range.

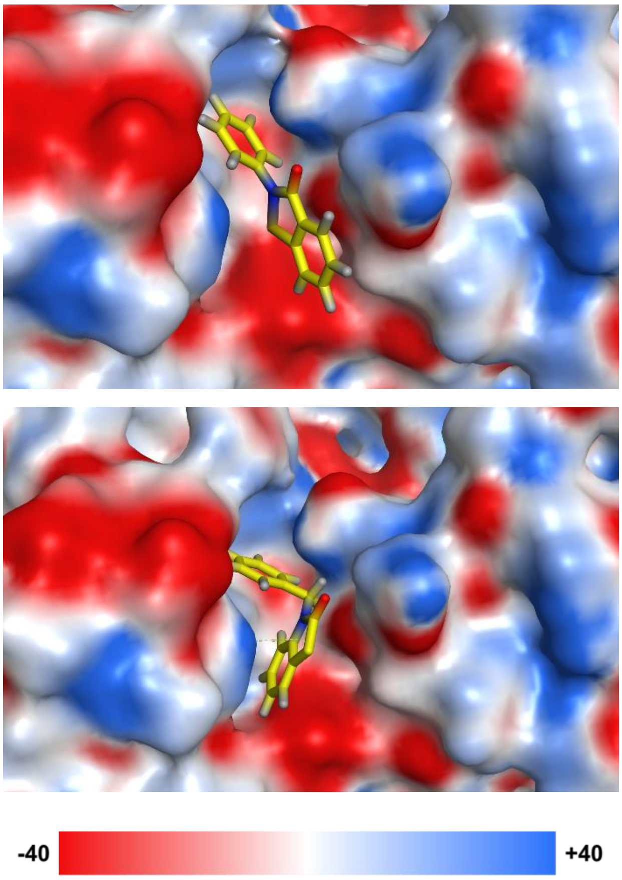

Figure S2. Docking results of ebselen (top) and compound **7** (bottom) in the region between domains II and III of M^{pro}. The protein is colored based on the electrostatic surface potential, computed using MOE 2019. The charge density is obtained by solving the Poisson-Boltzmann equation for the system. Significant patches are established by cutting the surface along iso-contour lines of absolute field value equal to 40 kcal mol^{-1} C^{-1}, keeping regions above. A default minimal patch area of 40 $Å^2$ filters out smaller, less relevant, patches. Potential values outside the range are clipped to the chosen range.
